# Successful Management of Recurrent Cholecystitis Four Years After Endoscopic Gallbladder Stenting (EGBS) Placement Using Internal and External Fistula Drainage

**DOI:** 10.7759/cureus.100481

**Published:** 2025-12-31

**Authors:** Takuya Koizumi, Akinori Maruta, Yosuke Ohashi, Takuji Iwashita, Masahito Shimizu

**Affiliations:** 1 First Department of Internal Medicine, Gifu University Hospital, Gifu, JPN; 2 Department of Gastroenterology, Shiga University of Medical Science, Gifu, JPN

**Keywords:** endoscopic retrograde cholangiopancreatography, endoscopic transpapillary gallbladder drainage, internal and external drainage, permanent endoscopic gallbladder stenting, recurrent cholecystitis

## Abstract

Recurrent cholecystitis can occur even after permanent endoscopic gallbladder stenting (EGBS), and optimal management in such situations remains challenging. In the present case, endoscopic transpapillary gallbladder drainage (ETGBD) was successfully achieved by using the indwelling EGBS to facilitate guidewire access to the gallbladder. Endoscopic nasobiliary gallbladder drainage was also placed, enabling combined internal and external drainage. The patient experienced a favorable clinical course following this approach. This case suggests that ETGBD is a feasible therapeutic option for recurrent cholecystitis after permanent EGBS placement, with the pre-existing stent aiding in gallbladder cannulation. Combined drainage may be particularly advantageous in the setting of purulent bile to ensure effective decompression.

## Introduction

Laparoscopic cholecystectomy is the standard treatment for acute cholecystitis, as recommended by the Tokyo Guidelines 2018 [1]. However, in high-risk surgical patients who are refractory to conservative treatment, gallbladder drainage is required to control the infection. Gallbladder drainage may necessitate long-term indwelling catheterization. Currently, three approaches are available: percutaneous transhepatic gallbladder drainage (PTGBD), endoscopic transpapillary gallbladder drainage (ETGBD), and endoscopic ultrasound-guided gallbladder drainage (EUS-GBD) [2-4].

PTGBD is a well-established technique but is contraindicated in patients with coagulopathy, massive ascites, or anatomical difficulties such as Chilaiditi syndrome [5]. Moreover, permanent PTGBD tube placement is generally avoided due to concerns regarding quality of life (QOL). ETGBD has been reported in numerous studies as a useful and safe alternative treatment to PTGBD [5-7]. Endoscopic gallbladder stenting (EGBS) allows for internal fistula management, making permanent stent placement a viable treatment option in high-risk surgical patients. Although permanent EGBS placement can prevent long-term recurrence of cholecystitis, most follow-up periods in existing reports are less than four years. Moreover, it has been reported to be associated with late adverse events (AEs), such as cholangitis and gallbladder perforation [8,9]. EUS-GBD is another treatment option for high-risk surgical patients; however, the availability of dedicated devices is limited, and the procedure is not yet supported by well-established evidence. There is no established treatment strategy for recurrent cholecystitis in high-risk surgical patients after long-term placement of EGBS. Herein, we report a rare case of recurrent cholecystitis that developed four years after EGBS placement and was successfully managed with internal and external fistula drainage, using the obstructed EGBS as a *guide* to straighten the cystic duct and employing combined endoscopic nasobiliary gallbladder drainage (ENGBD) and a new EGBS for severe purulent cholecystitis.

## Case presentation

**Figure 1:**
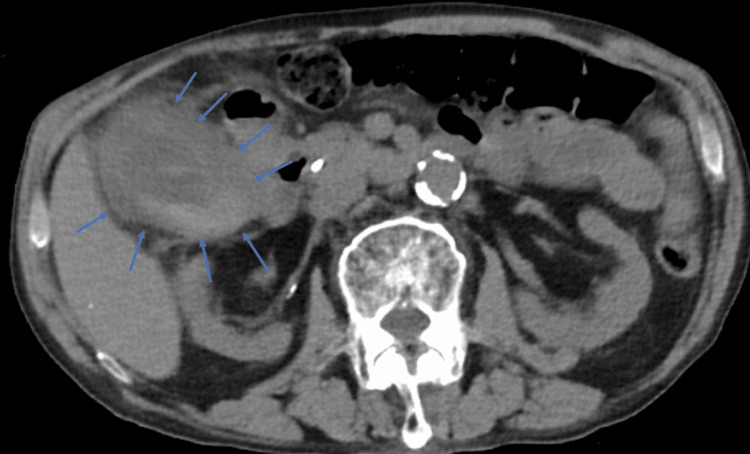
Abdominal computed tomography image showing gallbladder wall thickening and increased pericholecystic fat density. The blue arrow indicates the gallbladder wall.

**Figure 2:**
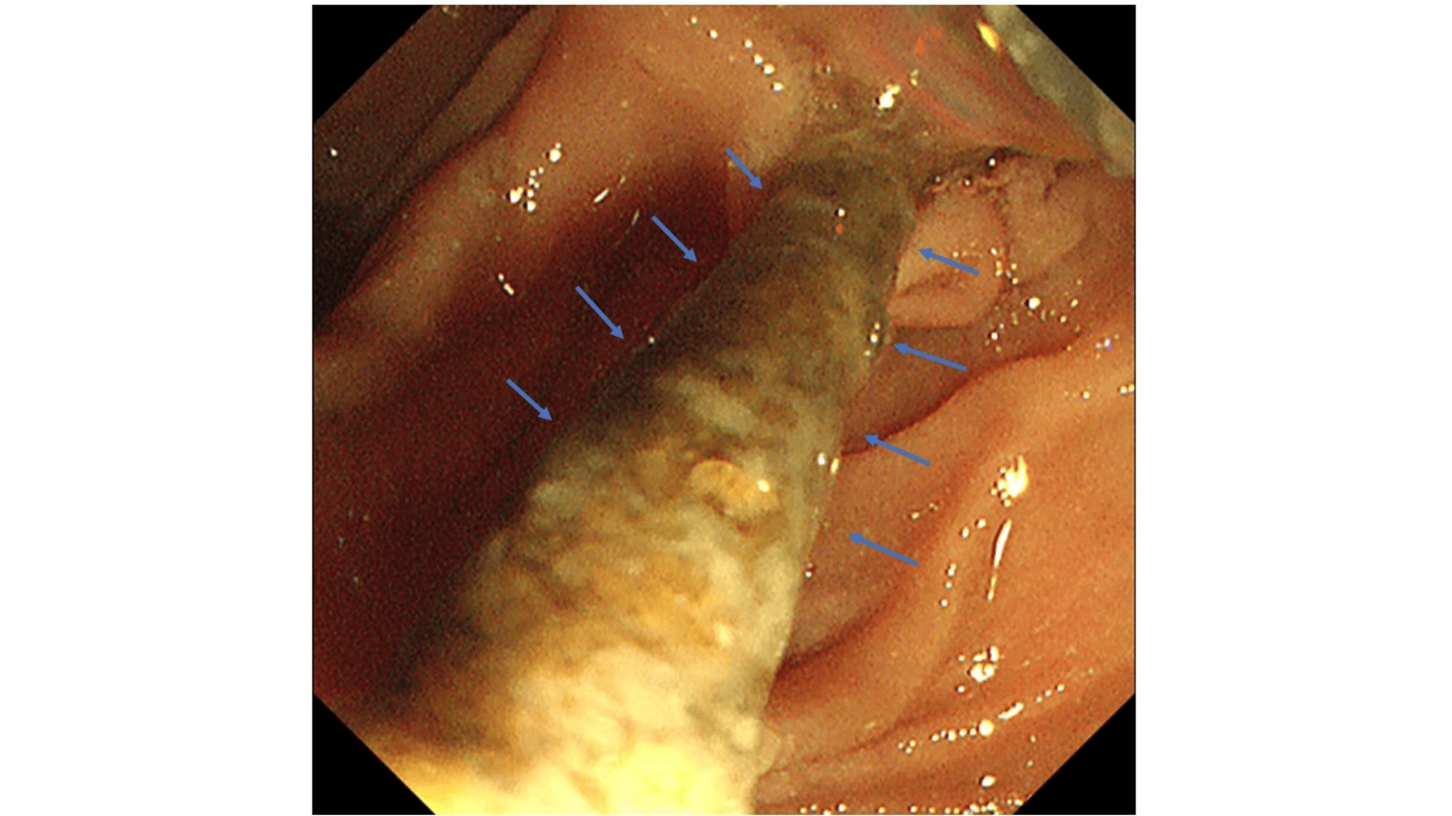
Endoscopic image showing occlusion of the previously placed EGBS by debris. The blue arrow indicates the previously placed EGBS. EGBS, endoscopic gallbladder stenting

An 83-year-old man had undergone EGBS for cholecystitis four years ago. Because cholecystectomy was considered high risk due to multiple comorbidities, including a history of abdominal aortic aneurysm surgery and cerebellar infarction, as well as ongoing treatment with aspirin and edoxaban, permanent EGBS placement was selected as a preventive measure against recurrence of cholecystitis. Since then, he had not experienced any recurrence. He later presented to our emergency department with a performance status (PS) of 2, complaining of abdominal pain. His vital signs were as follows: body temperature, 38.6 ℃; blood pressure, 124/56 mmHg; pulse, 86 beats/min; and oxygen saturation, 95% on room air. Physical examination revealed tenderness in the right costal region; however, the abdomen was soft with no rebound tenderness. The laboratory examination results are summarized in Table 1. Elevated hepatobiliary enzyme levels and inflammatory markers were observed. Non-contrast abdominal computed tomography revealed gallbladder wall thickening, increased pericholecystic fat density, and an impacted gallstone in the gallbladder neck (Figure 1). No dislodgement of the EGBS was observed. Based on these findings, we suspected that gallstone impaction in the gallbladder neck, along with obstruction of the EGBS, led to acute calculous cholecystitis. Given the concurrent renal dysfunction, the patient was diagnosed with severe (Grade III) acute cholecystitis according to the 2018 Tokyo criteria, and urgent gallbladder drainage was deemed necessary. As the patient was receiving edoxaban and aspirin, PTGBD was considered high risk due to bleeding concerns; therefore, emergency ETGBD was performed on the same day, as it does not require procedures associated with a high risk of bleeding. Upon insertion of the endoscope into the duodenal papilla, endoscopic examinations revealed that the previously placed EGBS was occluded with debris (Figure 2). Cannulation of the bile duct was performed using a G-Cannula (Gadelius Medical, Tokyo, Japan), and a 0.025-inch guidewire (VisiGlide 2; Olympus Medical Systems, Tokyo, Japan) was advanced into the gallbladder alongside the existing EGBS. The previously placed EGBS facilitated relatively easy guidewire advancement into the gallbladder.

**Table 1:** Laboratory examination. AST, aspartate aminotransferase; ALT, alanine aminotransferase; ALP, alkaline phosphatase; LDH, lactate dehydrogenase; γ-GTP, gamma-glutamyl transpeptidase; BUN, blood urea nitrogen

Parameter (unit)	Day 1 (Admission)	Day 2	Day 3	Reference range
WBC (×10³/μL)	9.23	3.55	3.28	3.3-8.6
RBC (×10⁶/μL)	2.76	2.36	2.56	4.3-5.5
Hemoglobin (g/dL)	8.0	6.9	7.5	13.7-16.8
Platelet count (×10³/μL)	191	149	180	158-348
Prothrombin time (PT) (seconds)	24.9	25.7	17.5	9.6-13.1
PT-INR	2.20	2.27	1.53	0.8-1.2
Activated partial thromboplastin time (APTT) (seconds)	33.6	33.1	29.5	24-34
Fibrin degradation products (FDPs) (μg/mL)	8.3	10.4	12.9	<5.0
D-dimer (μg/mL)	3.6	NA	5.3	<1.0
Total protein (g/dL)	6.3	5.4	5.4	6.6-8.1
Albumin (g/dL)	2.5	2.0	2.0	4.1-5.1
AST (U/L)	1307	1136	414	13-30
ALT (U/L)	329	523	364	10-42
ALP (U/L)	414	382	334	38-113
LDH (U/L)	414	895	229	124-222
γ-GTP (U/L)	209	210	196	13-64
Total bilirubin (mg/dL)	1.4	1.5	1.3	0.4-1.5
Direct bilirubin (mg/dL)	1.0	1.2	0.8	0.0-0.4
Creatinine (mg/dL)	2.16	2.44	2.19	0.65-1.07
BUN (mg/dL)	47.0	51.5	38.9	8.0-20
Sodium (mmol/L)	137	138	138	138-147
Potassium (mmol/L)	4.3	4.3	4.2	3.6-4.8
Chloride (mmol/L)	103	104	108	101-108
C-reactive protein (CRP) (mg/dL)	14.4	13.5	9.47	<0.14
Procalcitonin (ng/mL)	18.4	NA	NA	<0.05

**Figure 3:**
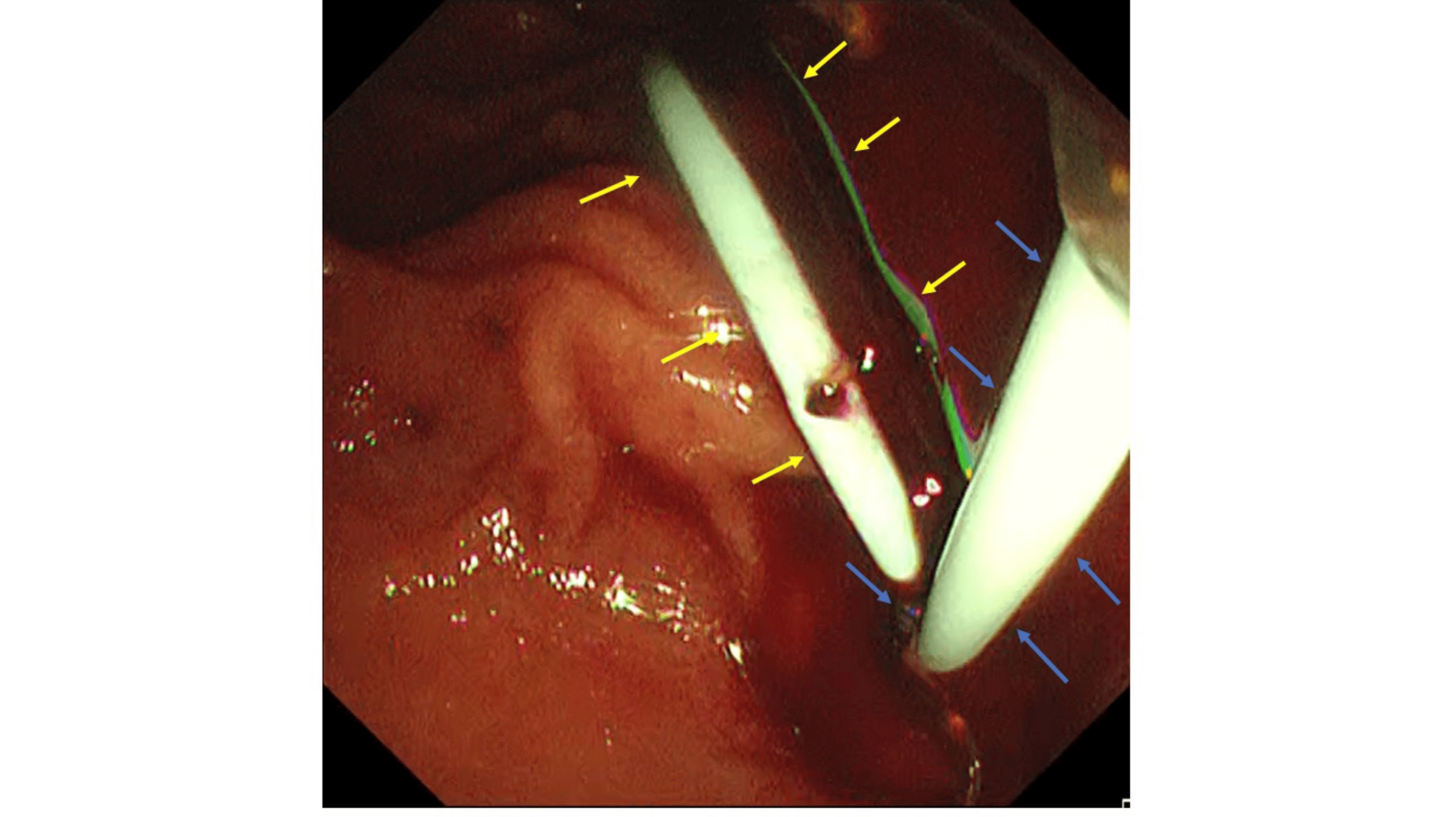
Endoscopic image showing placement of the EGBS and ENGBD tube. Blue arrows indicate the ENGBD, and yellow arrows indicate the EGBS. EGBS, endoscopic gallbladder stenting; ENGBD, endoscopic nasobiliary gallbladder drainage

**Figure 4:**
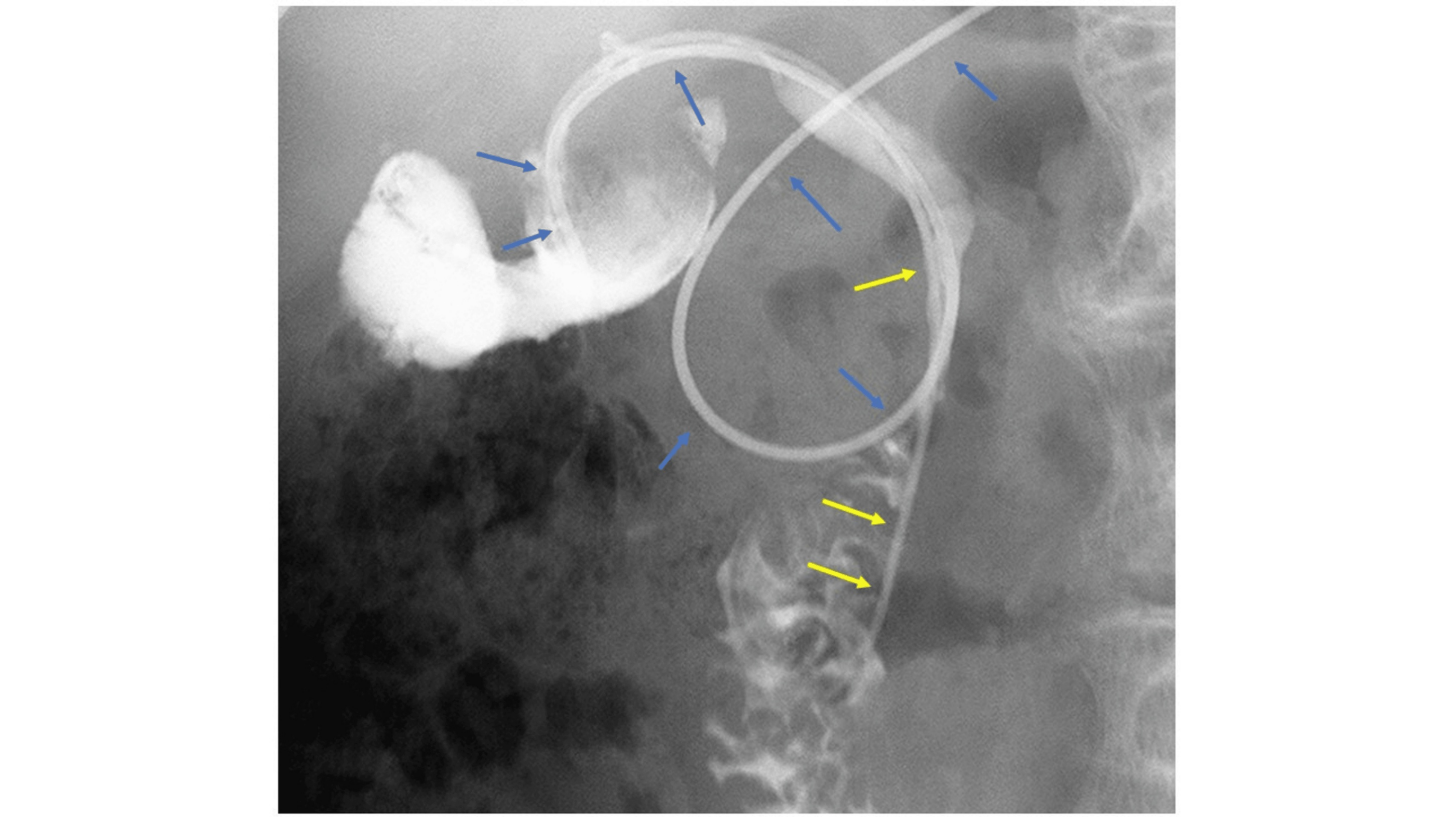
Cholangiography through the ENGBD tube showing contrast passage into the duodenum. Blue arrows indicate the ENGBD, and yellow arrows indicate the EGBS. EGBS, endoscopic gallbladder stenting; ENGBD, endoscopic nasobiliary gallbladder drainage

After advancing the guidewire into the gallbladder, the previously placed EGBS was then removed using grasping forceps. Subsequently, a double-lumen catheter (Uneven catheter; Piolax Medical Devices, Kanagawa, Japan) was inserted, and an additional 0.025-inch guidewire (J-WIRE; J-MIT, Shiga, Japan) was inserted into the gallbladder. Bile was aspirated through the catheter, confirming the presence of hemorrhagic purulent bile. Based on the characteristics of the bile, internal drainage alone was considered to carry a high risk of stent occlusion and inadequate drainage. External drainage, which enables continuous drainage, monitoring of bile output, and irrigation as needed, was therefore deemed preferable. However, because external drainage alone carried a risk of accidental self-removal, internal drainage was additionally performed to enhance drainage efficacy and to reduce the risk of recurrence after resolution of cholecystitis. A new EGBS (IYO stent; Gadelius Medical, Tokyo, Japan) and an endoscopic nasobiliary gallbladder drainage (ENGBD) tube (NB-Braid, 5Fr; Piolax Medical Devices, Kanagawa, Japan) were placed to complete the procedure (Figure 3). No significant adverse events (AEs) were observed during or after the procedure. (Escherichia coli was detected in the bile culture.) Intravenous tazobactam/piperacillin (2.25 g every 8 hours) was initiated on the day of admission and continued until hospital day 9. The minimum inhibitory concentration (MIC) of tazobactam/piperacillin was ≤2 μg/mL, and the isolate was susceptible. Therefore, the selection of this antibiotic regimen was considered appropriate. On hospital day 2, the patient became afebrile, and laboratory parameters, including liver enzymes and inflammatory markers, showed improvement on blood examinations performed on hospital days 2 and 3. The drainage from the ENGBD tube was approximately 60 mL/day and green-serous in appearance. On hospital day 4, cholangiography through the ENGBD tube showed contrast passage into the duodenum, suggesting resolution of gallstone impaction (Figure 4), and clamping of the tube was initiated.

The patient continued to show no cholecystitis recurrence, and the ENGBD tube was removed on hospital day 6. He continued to remain symptom-free and was discharged on hospital day 10. Given the patient's advanced age and multiple comorbidities, cholecystectomy was considered a high-risk procedure. Additionally, neither the patient nor his family desired surgical intervention. Therefore, a decision was made to permanently replace the EGBS without performing a cholecystectomy.

## Discussion

The efficacy and safety of permanent EGBS have been investigated as a therapeutic option for high-risk surgical patients. Several studies have reported a lower cholecystitis recurrence rate using this approach [6,10]. In a retrospective study by Maruta et al., high-risk surgical patients with acute cholecystitis were divided into a group that received permanent EGBS placement (*n* = 40) and a group in which the drainage tube was removed after initial gallbladder drainage (*n* = 131). The median follow-up periods were 375 days and 307 days, respectively. Cholecystitis recurred in 2 patients (5%) in the permanent EGBS group and in 25 patients (19.1%) in the tube removal group, showing a significantly lower recurrence rate in the EGBS group (*P* = 0.024) [8].

In another retrospective study by Inoue et al., high-risk surgical patients with acute cholecystitis were divided into permanent EGBS (*n* = 297) and PTGBD groups (*n* = 231). The technical success rates were 75.4% and 98.7%, respectively, with the permanent EGBS group showing a significantly lower success rate (*P* < 0.001). The median follow-up periods were 1,115 and 1,136 days, respectively. Cholecystitis recurrence occurred in 10 patients (6.3%) in the permanent EGBS group and in 23 patients (19.2%) in the PTGBD group, with a significantly lower recurrence rate in the permanent EGBS group (*P* = 0.001) [9]. These study results demonstrate the effectiveness of permanent EGBS placement in suppressing the recurrence of cholecystitis. In this case, the patient remained free from recurrent cholecystitis for four years following permanent EGBS placement. This clinical course is consistent with previously reported evidence suggesting that long-term EGBS placement can suppress recurrence of cholecystitis. However, there are few published reports describing follow-up periods exceeding four years after EGBS placement, and this represents one of the novel aspects of the present case.

The mechanism underlying the lower recurrence rate is believed to involve not only the patency of the stent but also its mechanical role in preventing gallstone impaction at the cystic duct or gallbladder neck. Additionally, the stent facilitates the *wicking* of bile from the gallbladder to the duodenum along its outer surface through capillary action, thereby reducing the risk of cholecystitis [11,12]. To the best of our knowledge, there have been no previous reports describing patients followed for as long as four years after permanent EGBS placement. In this context, the present case is noteworthy in that cholecystitis recurred after a prolonged recurrence-free period of four years following EGBS, highlighting the novelty of late recurrence after permanent EGBS. In the present case, recurrent cholecystitis was considered to be caused by an impacted gallstone in the gallbladder neck and obstruction of the previously placed EGBS due to debris accumulation, as the endoscopically placed stent lumen was found to be occluded with residual debris.

However, permanent EGBS placement also has disadvantages. As the observation period for permanent EGBS placement has lengthened, sporadic reports of AEs have emerged, including recurrent cholecystitis and other late AEs. In the aforementioned study by Inoue et al., late AEs (excluding cholecystitis) were observed in 31 of 158 patients (19.6%) in the EGBS group [9]. These included cholangitis in 27 patients (17.1%), gallbladder perforation in two patients (1.3%), liver abscess in one patient (0.6%), and pleuritis in one patient (0.6%). In contrast, 13 of 120 patients (10.8%) in the PTGBD group experienced late AEs, excluding cholecystitis, with the EGBS group showing a significantly higher rate of late AEs (*P* = 0.049). Prolonged bile duct stenting is considered to cause cholangitis due to bile stasis and duodenobiliary reflux. The pathogenesis of biliary stent occlusion is multifactorial and is thought to be caused by biofilm formation induced by food debris and bacteria.

Furthermore, in recent years, there have been reports on the long-term placement of drainage under EUS guidance. In a study by Belen et al., EUS-GBD was performed in 50 patients with acute cholecystitis who were considered high-risk surgical candidates. Lumen-apposing metal stents (LAMS) were permanently placed, and the patients were followed up for three years. AEs were evaluated retrospectively. Recurrent cholecystitis was observed in two patients (4%). AEs related to LAMS placement occurred in 14 patients (37.5%), including stent occlusion in three patients, stent burial in two, gastric outlet obstruction in two, and stent migration in seven [13]. These results suggest that EUS-GBD using LAMS is also useful in the management of cholecystitis in high-risk surgical patients; however, further verification is needed, including a comparison with EGBS.

Currently, there is no established treatment strategy for recurrent cholecystitis in high-risk surgical patients after long-term placement of EGBS. ETGBD is an effective treatment option for gallbladder drainage; however, its major limitation is its lower technical success rate compared to other drainage methods, such as PTGBD and EUS-GBD. Previous reports have indicated that anatomical factors such as cystic duct branching patterns significantly contribute to ETGBD failure [14-18]. In the present case, the pre-existing obstructed EGBS served as a guide and had straightened the cystic duct, thereby facilitating relatively easy advancement of the guidewire into the gallbladder. Furthermore, the presence of bloody purulent bile raised concerns regarding a high risk of occlusion if internal drainage alone were performed. Therefore, an additional external drainage tube was placed, enabling monitoring of the drainage output and irrigation as needed, which resulted in a favorable clinical course. Although no previous studies have described the combined use of internal and external fistula drainage for recurrent cholecystitis, dual EGBS placement has been reported. Sobani et al. retrospectively evaluated 21 high-risk patients who underwent dual EGBS for cholecystitis. Stents were placed either in a single session or in a staged approach at 4- to 6-week intervals. Due to the risk of bile duct or gallbladder perforation when guidewires were passed through inflamed tissues, most cases utilized a two-stage stenting approach. The technical and clinical success rates were 100%. AEs occurred in two patients (9.4%): one experienced stent migration, and one developed post-endoscopic sphincterotomy (EST) bleeding**.** During a median follow-up period of 341 days, cholecystitis recurrence was observed in only one patient (4.7%), who was subsequently treated with EUS-GBD using LAMS. The placement of two stents appears to reduce the risk of obstruction, as bile can continue to flow through gaps even if one stent becomes occluded, thereby prolonging the interval to recurrence [19]. Based on previous reports and the clinical course of the present case, ETGBD may be considered an effective treatment option for recurrent cholecystitis following permanent EGBS placement because the existing stent can serve as a guide, facilitating relatively easy advancement of the guidewire into the gallbladder. Furthermore, in patients at a high risk of stent occlusion, such as those with bloody purulent bile, the use of multiple drainage routes may be beneficial. Importantly, unlike previously reported dual stents cases, our case uniquely utilized an obstructed pre-existing EGBS as a functional guide to straighten the cystic duct and enabled the combined use of ENGBD and a newly placed EGBS for effective drainage.

In contrast, Higa et al. conducted a retrospective comparison between EUS-GBD and ETGBD in high-risk surgical patients with acute cholecystitis. Among 39 patients in the EUS-GBD group and 32 in the ETGBD group, the recurrence rates of cholecystitis during the median follow-up periods of seven and five months were 2.6% (1 patient) and 18.8% (6 patients), respectively, indicating a lower recurrence rate in the EUS-GBD group [20].

An unresolved issue is the lack of an established treatment strategy for recurrent cholecystitis after permanent EGBS placement. In cases of recurrent cholecystitis after EGBS placement, treatment with EUS-GBD may be a viable option, and management strategies should be determined based on individual patient characteristics. In the present case, the patient was receiving dual anticoagulant therapy; therefore, PTGBD and EUS-GBD were considered to carry a high risk of bleeding. In addition, because an EGBS had been placed previously, ETGBD was considered to have a high likelihood of technical success, and drainage via ETGBD was therefore selected. Although the evidence regarding outcomes and complications after reintervention by replacement of EGBS remains limited, long-term EGBS placement is expected to reduce the recurrence of cholecystitis. However, continued vigilance is required for late AE other than recurrent cholecystitis, such as cholangitis and gallbladder perforation.

## Conclusions

In conclusion, we encountered a patient in whom EGBS was initially effective in managing acute cholecystitis; however, recurrence occurred after a four-year interval. In cases involving permanent EGBS placement, the potential for late AEs exists, highlighting the need for careful long-term follow-up. A unique aspect of this case is that obstruction of the previously placed EGBS paradoxically facilitated guidewire access by straightening the cystic duct, thereby allowing relatively easy advancement of the guidewire into the gallbladder, resulting in successful ETGBD. Additionally, by employing both internal and external drainage management, we were able to enhance the drainage efficiency and achieve a favorable clinical course. This case suggests that ETGBD is an effective treatment option for recurrent cholecystitis after permanent EGBS placement. However, this report is limited by its single-case design, and further accumulation of cases and long-term data is required to validate the generalizability of these findings.
